# Changes in Electrical Conductance of Polymer Composites Melts Due to Carbon Nanofiller Particles Migration

**DOI:** 10.3390/polym13071030

**Published:** 2021-03-26

**Authors:** Oleg V. Lebedev, Galina P. Goncharuk, Alexander N. Ozerin

**Affiliations:** 1 Moscow Institute of Physics and Technology, Institutsky lane 9, Dolgoprudny, 141700 Moscow, Russia; 2 Enikolopov Institute of Synthetic Polymer Materials of RAS, Profsoyuznaya st. 70, 117393 Moscow, Russia; ggoncharuk@ispm.ru (G.P.G.); ozerin@ispm.ru (A.N.O.)

**Keywords:** nanocomposite preparation, carbon nanomaterials, migration, nanocomposite electrical conductivity, polypropylene

## Abstract

In this work, the results of investigation of the effect of polymer composite melts electrical conductance increase with time are presented. The conductance time dependencies were obtained for composites based on polypropylene filled with carbon nanoparticles of different types. The dependencies were analyzed to demonstrate the possibility of correlation of the conductance kinetics with different composite parameters, such as the filler geometry. Additional studies were carried out, such as electron microscopy study, conductance measurements after consecutive surface layer removal, and composite melt conductance measurements using a three-electrode scheme. The results showed that the increased electrical conductance of the composite materials can be attributed to the formation of an enriched with the filler particles surface layer, which happens during the stay of the composite in a melt state. Analysis of the experimental data, along with the results of numerical modeling, allowed to suggest a possible filler distribution transformation scheme. The physical premises behind the investigated effect are discussed.

## 1. Introduction

One of the popular areas of research nowadays is investigation of the process of filler nanoparticles migration to the interface between multiple phases in polymer composite systems processed in a melt state. The nanoparticles can effectively stabilize the interface by decreasing the surface tension. Different research works demonstrate presence of the phenomenon of migration of the filler particles to the phase-to-phase interface, particularly for carbon nanoparticles of different types acting as the stabilizers. Unfortunately, the results of these works do not include analysis of the kinetics of the migration process, since, most of the time, the information on filler particles distribution transformation is obtained from the analysis of electron microscopy data for the composite materials thin cuts [1,2,3,4].

Bose et al. investigated whether phase separation in polymer blends can be used as a tool to create well dispersed conducting filler rich domains in 3D with controlled morphology, potentially resulting in more effective percolation [1]. According to the electron microscopy data, the phase separation resulted in a heterogeneous distribution of NH_2_- multiwalled carbon nanotubes (MWCNTs) in poly(α-methyl styrene)-*co*-acrylonitrile)/poly(methyl methacrylate) blends. It was assumed that the migration of NH_2_-MWCNTs is controlled by the thermodynamic forces that drive phase separation and led to an increase in their local concentration in a specific phase resulting in percolative “network-like” structure.

Xiong et al. in their work [4] observed migration of MWCNTs from polycarbonate (PC) phase to the acrylonitrile-butadiene-styrene (ABS) phase when PC/MWCNT masterbatch was diluted with PC and ABS by melt mixing for 5 min with 70% of ABS. The migration was explained by a combination of the morphology evolution and higher affinity of MWCNTs to polybutadiene than to PC.

Alig et al. in their work [5] studied electrical conductivity of the melts of the composites based on polypropylene (PP) filled with different amounts of the MWCNTs after the extruder was stopped and during the cooling cycle. For a composite sample containing 2 wt.% of MWCNTs the direct current (DC) conductivity demonstrated significant increase with time after the shear deformation was removed. This effect was attributed to the reorganization of the conducting network made of the filler particles, which was initially disturbed by the shear deformation applied to it in the extruder. Time dependence of the conductivity was explained by the dynamic agglomeration of the nanoparticles. Continuing the work [5], Alig et al. investigated kinetics of the electrical conductance recovery for the PC-based composite melts, filled with MWCNTs, after application of the shear deformation to the composite system [6]. Similarities between conductivity kinetics, obtained for the first and second conductance recovery cycles, were observed. At the same time, loss of the initially fine dispersion of MWCNTs in the composite volume due to MWCNTs agglomeration was noted.

Deng et al., in their publication [7], discuss the reasons behind electrical conductivity changes for polymer composite materials, filled with MWCNTs and carbon black (CB) particles, during their stay at the isothermal conditions at temperatures above melting point of the base polymers for different periods of time. Particularly, the conductivity increase was explained by the formation of the conducting fiber-like structures made of the growing filler particles agglomerates. To model the process of MWCNTs clusters agglomeration, a second-order kinetic equation was used, initially proposed by Heinrich et al. [8], for modeling of the formation of the conducting structures in elastomers. Solution of this kinetic equation gives the following relation between volume fraction of the growing agglomerates and time:

(1) $$c_{A}\left(t\right)=c_{A0}+\left(c_{A\infty }-c_{A0}\right)\cdot \left(1-\;\frac{1}{1+4kt\left(c_{A\infty }-c_{A0}\right)}\right),$$

where *t*—time, $c_{A0}$ and $c_{A\infty }$—values of initial (*t* = 0) and final (*t* *→* ∞) volume fractions of agglomerates, respectively, *k*—rate constant.

To model a composite material electrical conductivity dependence on the filler concentration, the semi-empirical Fournier model [9] is often used. The model is based on the Fermi–Dirac distribution and describes insulator–conductor transition in a composite material. Taking into account the Equation (1), the corresponding modified Fournier equation can be written as:

(2) $$\log g_{DC}\left(t\right)=\log g_{0A}+\frac{\log g_{0M}-\log g_{0A}}{1+exp\left[b\left(c_{A}\left(t\right)-c_{c}\right)\right])},$$

where $g_{DC}$—*DC* electrical conductivity of the composite material, $g_{0A}$ и $g_{0M}$—conductivity of a MWCNTs agglomerate and the polymer matrix, respectively, $c_{c}$—percolation threshold, b—empirical parameter determining the conductivity behavior close to the percolation threshold.

The relation (2) demonstrated satisfying approximation quality for the experimental conductivity-time curves obtained for PP/CB composites melts, as it was reported in [7]. Unfortunately, the relation (2) contains a large number of parameters, while the conductivity, according to the assumptions made in [9], is dependent solely on the nanoparticles agglomeration degree. Thus, the modeling approach based on this equation cannot be considered as a universal.

In the work of Pegel et al. [10] mobility of commercially available MWCNTs in PC melts was studied. It was demonstrated that Van der Waals interactions between MWCNTs in a composite melt results in different degrees of MWCNTs agglomeration during processing of the composite in an extruder. It was concluded that by using low processing temperatures, thus lowering the filler particles mobility in the composite melt volume, a better dispersion of the MWCNTs can be achieved. Additionally, an increase in the percolation threshold, with an increase in the MWCNTs agglomeration, was observed. At the same time, a controversial result was obtained for electrical conductivity, which increased in case if the composite samples were obtained by hot molding at low molding rate. This effect was attributed to some “secondary” agglomeration process occurring in the course of the stay of the composite material in a melt state.

In their review paper, Salehiyan et al. [11] present a detailed fundamental study of influence of the conductive nanoparticles migration process on the filler distribution for composites based on a mixture of multiple mutually immiscible polymers. It was demonstrated that the spreading coefficient is the main thermodynamic parameter that determines the distribution of nanoparticles during composite processing. It is also noted that the intensity and the duration of the mixing of a multiphase polymer system, as well as the viscosities of each phase, and a size and a shape of the nanoparticles, can play a significant role in determining the final distribution of the filler particles. Moreover, it was shown that the migration rate depends on the chemical composition of the nanoparticle surface, as well as the viscosity of the polymer phases. In addition, the nanoparticles with a high aspect ratio migrate much faster. It was found that in composites with “double percolation”, which are based on the immiscible polymer components and characterized by low percolation threshold values, the filler particles are localized at the interface between the polymer phases. Additionally, in the review the results of studies of the kinetic dependences of the electrical conductivity of composite melts are analyzed. It is shown that most of the research groups assume that the nanoparticles remain at the interface between polymer phases. According to them, the changes in the electrical conductivity are due to a transformation of the spatial distribution of the polymer components, accompanied by a decrease in the specific surface area of the interface.

Teng et al. used molecular dynamics (MD) methods to study the athermal process of segregation of nanoparticles of simple geometries (spherical and cubical) in a thin film formed by a mixture of the particles and polymer molecules [12]. Due to the presence of the polymer molecules, enrichment with nanoparticles of the near-surface layer of the film at the low nanoparticles contents was noted. This effect is consistent with the theory of depletion forces [13,14,15]. In the case of a relatively large number of nanoparticles, the opposite result was obtained: the particles fully agglomerated in the bulk of the film. At the intermediate nanoparticles content, at some point of time they formed a percolation cluster in the bulk, connecting the enriched with the nanoparticles surface layers of the thin film. Because of the athermal nature of the studied system, which implies segregation solely due to the entropy factor, it is not possible to estimate how the system would behave if there was an enthalpy component between the various components of the system. At the same time, researchers dealing with modern models of depletion forces note the necessity of the enthalpy factor consideration for correct interpretation of the experimental data [14].

The results of experimental studies by Grekhov et al. [16] demonstrate that the concentration of MWCNTs in a solvent is a critical parameter determining stability of the dispersion. The kinetics of agglomeration of MWCNTs dispersed in chloroform were investigated by optical spectroscopy and dynamic light scattering. The critical concentration of nanotubes was determined at which no sedimentation and agglomeration processes were observed. The obtained concentration value was orders of magnitude lower than the value corresponding to the percolation threshold for MWCNTs, calculated according to the classical percolation theory [17,18,19,20].

In the work of Zhang et al. [21], the authors demonstrated construction of highly efficient electrical conductive networks in CB-filled polyoxymethylene–thermoplastic polyurethane–polyamide 6 (PA6) ternary blends through the formation of a hierarchical structure composed of a minor PA6 phase as droplets inside one major phase (TPU) and CB particles localized at the TPU–PA6 interface by thermodynamically induced self-assembly. It was shown, that the obtained hierarchical structure can be thermodynamically predicted on the basis of the minimization of total interfacial energies, which was confirmed by the electron microscopy results. In addition, the hierarchical structure improved the composite thermal stability in comparison with the simple structure formed by the homogeneously dispersed CB particles.

In the pioneering works of Sumita et al. studied the dynamic processes of percolation network formation (dynamic percolation) for various polymer composites by tracing, in real time, the time dependence of electrical resistivity during isothermal treatment of composites at temperatures above melting temperature for such fillers as CB [22,23,24] and MWCNTs [24]. The authors introduced the concept of percolation time, corresponding to a certain heat treatment time when the electrical resistivity starts to significantly decrease. The results of the studies also suggested that the dynamic percolation could be used to trace the formation of filler conductive network and estimate the interaction between the filler particles and polymer molecules.

The study of the processes of self-assembly of carbon nanotubes (CNTs) dispersed in different polymer phases using the method of MD is described in [25]. Additionally, using the methods of MD, Gumerov et al. [26] simulated the segregation of micellar structures at the water–oil interface, which demonstrates the possibility for various systems to form saturated nanostructured monolayers under the influence of the enthalpy factor.

It should be noted that all the above-mentioned studies deal only with specific cases due to the limited ranges of studied model systems and verified experimental data. A detailed analysis of the scientific literature has shown that for polymer composites filled with carbon particles of different types at this moment no thorough studies of the evolution of the electrical conductivity of polymer composites melts were conducted considering possible significant contribution of the process of migration of the filler nanoparticles to the surface of the melts. At the same time, a study of a possibility of formation of a gradient structure in a composite material and controlling the composite properties both at the stage of initial formation and in the course of a subsequent thermal treatment is of undoubted scientific and practical interest. The possible area of applications can be very broad for such types of composites, which are classified as functionally graded materials [27]. Particularly, the gradient structure of the functionally graded composites can be effectively used for creation of sensors, electromagnetic interference (EMI) shielding components, capacitors, or components for different tribological applications [27,28,29].

The novelty of our work is in a comprehensive study of the process of segregation of the filler nanoparticles in the melts of polymer composites filled with carbon particles of different types. It is based on a thorough analysis of the polymer composite melts electrical conductance changes with time for a number of polymer composite systems. Our approach takes into account that the one of the most influencing segregation mechanisms can be migration of the nanoparticles to the surface of melts of the composite materials, which can be highly thermodynamically beneficial due to the minimization of total interfacial energies of the polymer composite components.

## 2. Materials and Methods

### 2.1. Materials

As a base polymer for the composite materials studied in this work, the commercially available amorphous-crystalline PP grade H030 GP/3 (SIBUR International GmbH, Vienna, Austria) was used [30].

Morphology of the filler is directly related to the mobility of the nanoparticles in a medium, as well as the its total surface energy in a medium [11]. Thus, it was decided to investigate polymer composites filled with carbon nanoparticles of different types, which, in perspective, should allow to understand the basic principles of the nanoparticles segregation in polymer composite melts.

**Figure 1:**
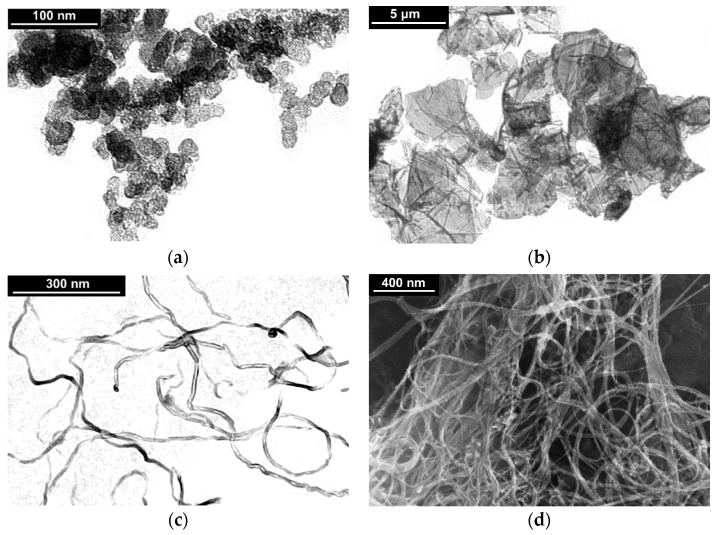
Results of transmission (**a**–**c**) and scanning (**d**) electron microscopy for the powders of various carbon nanoparticles used as the fillers ((**a**)—carbon black (CB); (**b**)—graphene nanoplatelets (GnPs)s; (**c**)—multiwalled carbon nanotubes (MWCNTs); (**d**)—single-walled carbon nanotubes (SWCNTs)).

One of the main fillers used in this work was CB, as the most commonly used filler with a shape close to a globular (Figure 1a). Particularly, the commercially available electrically conductive CB of brand P267-E (equivalent of N 472, Institute of Hydrocarbons Processing of the Siberian Branch of the Russian Academy of Sciences, Saint Petersburg, Russia) with specific surface area of 144 m^2^/g (according to the filler specification [31]) was chosen. Another type of filler used was graphene nanoplatelets (GnPs), characterized by a planar texture formed by several stacked layers of graphene. In this work, GnPs of commercial grade AO-3 (Graphene Laboratories, Calverton, NY, USA) with specific surface area of 80 m^2^/g (according to the filler specification [32]) (Figure 1b) were chosen. Finally, two types of CNTs were used: MWCNTs and single-walled carbon nanotubes (SWCNTs). The commercially available MWCNTs NC7000 (Nanocyl, Sambreville, Belgium) were chosen, as the most uniform in diameter and size (length ~1.5 μm, diameter ~9 nm, specific surface area 250÷300 m^2^/g), as well as characterized by high purity (according to the filler specification [33]) (Figure 1c). SWCNTs of the commercial grade Tuball (OCSiAl, Novosibirsk, Russia) with specific surface area of 331 m^2^/g (according to Krestinin et al. [34,35]) were selected, as the most affordable and widely used SWCNTs with a high level of electrophysical characteristics (Figure 1d). The results of a comprehensive study of the Tuball SWCNTs, performed by Krestinin et al., are presented in the publication [35].

### 2.2. Methods

#### 2.2.1. Composite Processing

Samples of composites were obtained via melt mixing of the polymer granules and nanoparticle powders in a micro-compounder (DACA Instruments, Goleta, CA, USA) designed to prepare compositions in laboratory quantities (up to 4 cm^3^). The quantity of the material in the micro-compounder chamber was calculated so the chamber was filled fully. The mixing procedure was carried out in air at 200 °C and 500 rpm for 10 min after the addition of the components into the compounder. The mixing was followed by granulation of a resulting composite strand of 2 mm in diameter to pellets of ~2 mm in length. The following notation is used further to indicate a composite of a certain composition: ‘polymer + ***X*** wt.% filler’, which means that the mixture contains ***X*** wt.% of the filler and (100—***X***) wt.% of the polymer. The formulation for some selected samples of composites is presented in Table 1.

**Figure 2:**
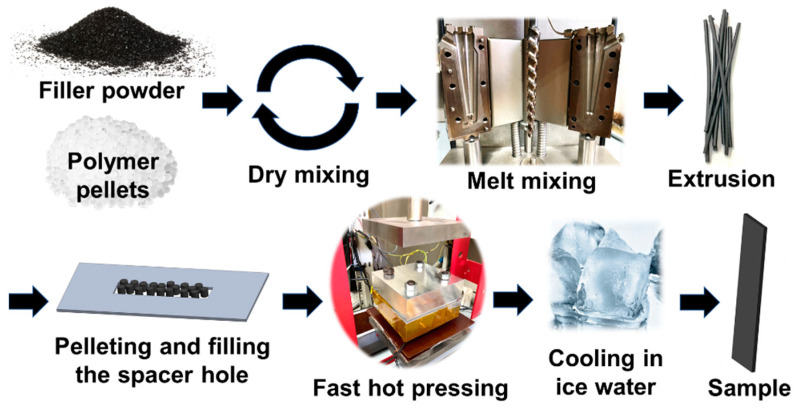
Scheme of manufacturing of solid composites samples for electrical conductivity measurements.

To determine the percolation thresholds for the composites filled with various types of the filler particles before their any additional thermal treatment, samples in the form of plates 12 mm × 60 mm × 1 mm in size were prepared from the granules of the composites by fast hot pressing (200 °C, pressure 3 MPa, time in a melt state <1 min). Cooling was performed by immersing the composite samples in ice water. The corresponding full composite processing scheme is presented in the Figure 2.

**Figure 3:**
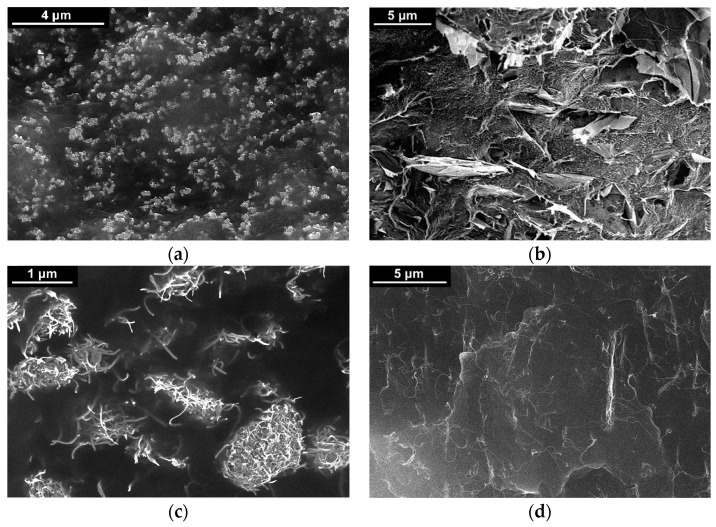
Results of scanning electron microscopy of low-temperature cleavages of composites based on PP filled with carbon nanoparticles with certain weight content: (**a**)—15 wt.% CB; (**b**)—10 wt.% GnPs; (**c**)—7.5 wt.% MWCNTs; (**d**)—0.5 wt.% SWCNTs.

To study the initial filler distribution and estimate size and shape of filler agglomerates in the composite materials of various compositions, electron microscopy studies of low-temperature cleavages of the composite samples were conducted (Figure 3).

#### 2.2.2. Methods of Investigation

DC electrical conductivity of the molded and cooled solid composite samples was measured using a four-probe resistance method via 34401A multimeter (Agilent, Santa Clara, CA, USA).

**Figure 4:**
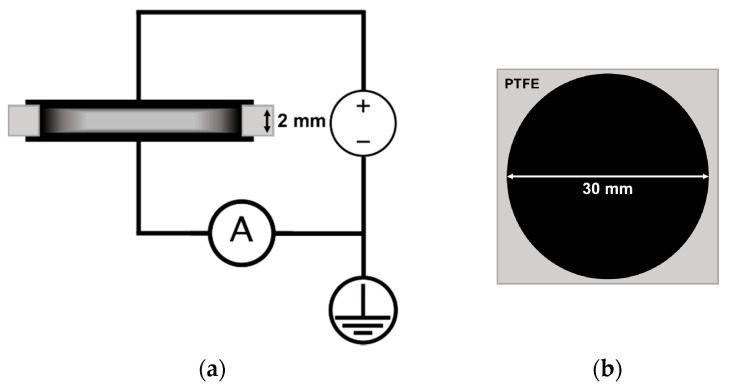
Experimental two-electrode circuit for measuring the electrical resistance of a composite melt: (**a**)—side view; (**b**)—top view.

To carry out kinetic measurements of the electrical resistance of the composite melts, granules of the prepared composite of various compositions were placed between two flat electrodes in a heated mold inside a circular hole of a spacer made of polytetrafluoroethylene (PTFE) (Figure 4). Required total amount of the granules was calculated based on the volume of the cavity formed by the PTFE spacer hole and the electrodes, taking into account the content of the filler in the composite and the density of the composite components. The composite granules were quickly heated to 205 °C, after which a pressure of 5 MPa was gradually applied to the composite melt. The temperature and pressure values were maintained for the duration of an experiment. A constant electrical voltage was applied between the electrodes during the entire experiment time. The voltage was provided by a highly stable AKIP 1147/1 power supply (Prist, Moscow, Russia). The standard preset value of the electric voltage was 2 V. The picoammeters 6485/E (Keithley, Cleveland, OH, USA) and A2-4 (MNIPI, Minsk, Belarus) were used for the current measurements. During an experiment, the values of the electric current between the electrodes were continuously recorded using a multichannel analog-to-digital converter E14-440 (L-Card, Moscow, Russia) with a frequency of 100 Hz.

As in the case of the composite samples studied to determine percolation threshold values, the cooling of the samples after the heat treatment was done by immersing the composite samples in ice water. The submersion of the samples in the water was performed along with the confining spacer, foil electrodes and polyimide isolating film, which prevented any contact of the sample with the water.

Electron microscopy studies of the carbon filler powders and the composite samples were performed using a Supra 50 VP LEO scanning microscope (Carl Zeiss AG, Oberkochen, Germany) with an INCA Energy + Oxford and Regulus SU8000 system for microanalysis (Hitachi, Ibaraki, Japan). The method of low-temperature cleavage at the temperature of liquid nitrogen was used to study samples of composites.

Viscosity of PP solutions in decalin at 135 °C was measured using the Ubbelohde viscometer on a base of IIRT-5 device (Tochmashpribor Ltd., Ivanovo, Russia) according to ISO 1628-3 standard. Characteristic PP viscosity [η] was estimated by extrapolation of the results to 0 solvent concentration.

To measure the profiles of the surfaces of the composites, a mobile profilometer-profilograph MarSurf M400 (Mahr, Esslingen, Germany) was used. The measurements were carried out on a 10 cm base for a measurement range of ± 25 µm with an accuracy of determining the height change of 0.8 nm and a probe movement speed of 1 mm/s.

#### 2.2.3. Modeling the Processes of Filler Migration to the Composite Surface

Numerical modeling was carried for composites containing CNTs using MD methods. An open source software package LAMMPS (Sandia National Laboratories, Albuquerque, NM, USA) was used as a modeling tool [36,37].

Several assumptions were made to create the representative composite volume elements. The first assumption was that what occurs in the bulk and in the near-surface layer depends weakly on the thickness of the composite at thicknesses higher than two lengths of a fully extended CNT. In this work, parameters of the MWCNTs NC7000 [33] were used to set the geometry of the simulated CNTs. According to this, the computational cell was represented as a parallelepiped 1.5 μm × 1.5 μm × 3 μm in size, which was confined in one direction (Z) by two walls characterized by an attractive potential, and was periodic in the other directions (X and Y).

As the attractive walls potential, the Lennard–Jones (L-J) potential was used. The L-J potential is widely used to characterize pair interatomic interactions, as well as to describe adsorption processes, due to its association with Van der Waals forces [38]. The polymer was represented in the form of a Langevin thermostat [39]. According to this concept, a CNT interacts with a polymer as with a thermal reservoir described by two components of the force: a randomly acting force $F_{r}$, and the friction force $F_{f}$. $F_{r}$ causes heating of CNTs and results from the fluctuation-dissipation theorem [38]. $F_{f}$ depends on the particles velocity and is required to prevent overheating of the particles.

CNTs were represented in the form of chains of rigid segments capable of free rotation around the joint points. This concept is based on the ability of CNTs to take assume various conformations, such as statistical coil. This is possible due to the large number of defects accumulated along the length of a CNT. A similar model, in which the so-called statistical segment (Kuhn’s segment) acts as a rigid link, is usually used to describe the dynamics of polymer chains [40]. The length of the statistical segment for the CNTs used in this work [33] was estimated at ~150 nm based on the data of electron microscopy of MWCNTs powder (Figure 1c), as well as the literature data [41].

To implement the chosen representation, each of the segments was represented as linearly aligned set of spherical particles. The sphericity was achieved by using the colloidal potential [42]. In this work, the Hamaker constant was varied to study the effect of the amplitude of the interaction force between CNTs on the studied processes.

Spherical particles in a CNT segment were positioned at a certain distance from each other, providing minimal longitudinal roughness of the segment. The spheres within one segment did not interact with each other, apart from the interaction formed due to the rigid fix. At the same time, the spheres of one segment were capable of interacting with the spheres of other segments of the same CNT, with segments of other CNTs, and the potentials of the confining walls. The number of spheres in a segment was determined by considerations of minimizing the segment roughness with a minimum number of spheres and the value of the CNT diameter [43]. For these purposes, the distance between adjacent spheres *l* was calculated using the formula:

(3) l=dCNT·cos(π6),

where $d_{CNT}$—CNT diameter.

CNTs were initially distributed in the rectangular volume element, preventing intersection between the segments. At the first step of the СNTs distribution creation, the initial points (points of one of the ends) of the CNTs were distributed evenly in the volume. The number of distributed CNTs was calculated taking into account specified CNTs volume fraction. From the initial points, CNTs were “grown” according to a simple procedure: at a distance corresponding to the length of one statistical segment, the next reference point was created, after which, at a random angle to the straight line formed by this point and the initial one, also at the length of one statistical segment, a new point was created. The procedure was repeated a number of times corresponding to the number of assumed statistical segments in the CNT plus one. The coordinates of the new points were limited by the walls along the Z coordinate of the volume element, from which the CNT segments were “reflected” into the depth of the volume. If the CNTs crossed the volume boundary in the X or Y direction, the growth of CNTs continued from the opposite wall. After distribution of all “skeletal” points of CNTs was completed, spherical particles described by the colloidal potential were distributed along the lines formed by the “skeletal” points.

**Figure 5:**
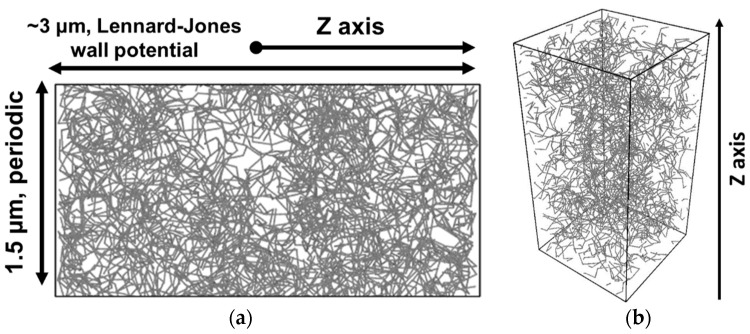
An example of a cell simulated by molecular dynamics (MD) methods with distributed carbon nanotubes (CNTs) with volume fraction of 0.5 vol%. (**a**)—side view; (**b**)—an oblique view.

An example of a simulated volume element with distributed CNTs 0.5 vol. % (~1 wt.%) is shown in Figure 5.

Numerical modeling was carried out for a set of CNTs content values (from 0.01 to 5 vol.%), temperatures, and values of the coefficients of the wall/CNT and CNT/CNT attractive potentials. For analysis of the CNTs distribution evolution in time, we used the histograms of the volume fraction of spheres plotted for the direction perpendicular to the planes of the confining walls (direction Z).

## 3. Results

### 3.1. Experimental Studies

At the first stage of the study, the composites based on PP with the addition of 0.5 wt.% SWCNTs were manufactured. For the obtained from the micro-compounder composites strands, their electrical conductivity was measured, which turned out to be below the sensitivity threshold of the multimeter used.

At the next stage, the strands of the composite were hot pressed for 15 min and cooled down at different rates: slow, intermediate, and fast. Slow cooling started with cooling to 170 °C at the rate of 2 °C/min, then it was followed by cooling to 140 °C at the rate of 1 °C/min, after which cooling to 100–115 °C was carried out at the rate of 2 °C/min. The resulting composite sample was characterized by resistivity of ~1 kOhm∙cm. The intermediate cooling was performed using a cold air flow to cool down the sample from 200 °C up to 100 °C in 15–20 min. The resulting composite sample was characterized by resistivity of ~0.5 kOhm∙cm. The last cooling rate was close to instantaneous: the sample was quickly immersed in ice water. The resulting composite sample was characterized by resistivity of ~1 kOhm∙cm. Since the order of magnitude of electrical conductivity of the composite samples was the same for every cooling rate studied, we consider the values to be in a range of error from each other, taking into account possible uncertainties in the wt.% of SWCNTs, heat treatment time, the quality of local dispersion, etc.

To establish the reasons behind the appearance of a high level of electrical conductivity of PP + 0.5 wt.% SWCNTs composites in comparison with the unmolded composite strands, it was decided to trace the behavior of the electrical conductivity of the material starting from the molding stage. Based on the results of the study, it was concluded that a high level of electrical conductivity of the material is achieved during the stay of the composite material in a melt state. In view of the fact that for a specific filler content in the composite, a change in the electrical conductivity of the material can only be associated with some kind of redistribution of electrically conductive particles in the bulk of the composite, and the fact that the electrical conductivity of the material monotonically increased during its stay in a melt state, it can be concluded that a filler segregation process is occurring in the composite melt.

For a detailed study of the effect of filler segregation in a polymer composite melt, polymer mixtures were prepared using nanoparticles of various types: CB, GnPs, MWCNTs, and SWCNTs. A study of the composites filled with nanoparticles of different geometry makes it possible to estimate the geometry influence on the segregation kinetics.

Due to the fact that in order to obtain a more noticeable change in the electrical conductivity of the material associated with the processes of segregation of the filler in the bulk of the composite melt, it is necessary to study a system, the conductivity of which would be initially very low. For that purpose, the concentration dependences of the electrical conductivity of materials were obtained for each type of filler. It is worth noting that in this work the effect of an increase in conductivity was also observed for composites, the initial filling degree of which exceeded the value of the percolation threshold, but the effect of changing the electrical conductivity was noticeably weaker in this case.

**Figure 6:**
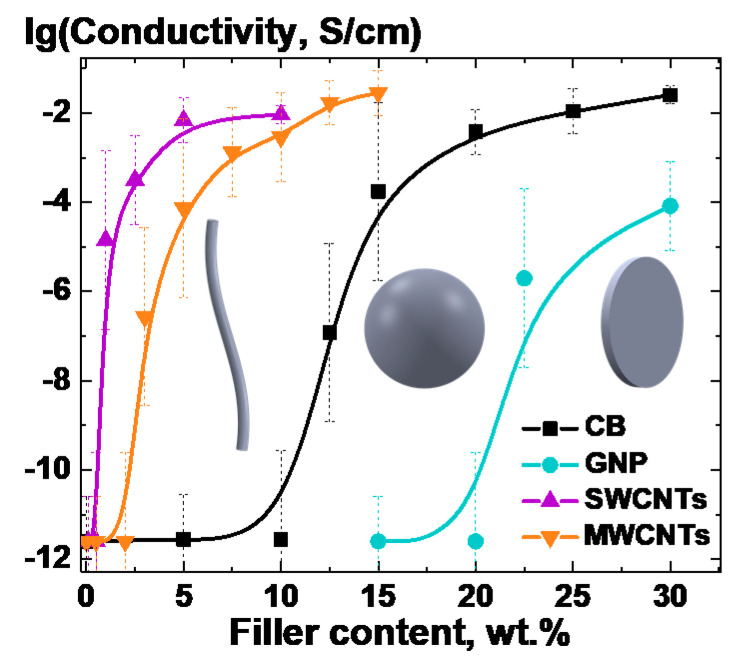
Dependences of the electrical conductivity of composites based on polypropylene (PP) on the content of various fillers (CB, GnPs, MWCNTs, SWCNTs) in the composite.

To exclude the effect of segregation process on the initial electrical conductivity of the samples, the thermal treatment time for the samples was kept minimal (<1 min), after which the composites were immersed into the ice water. The results of measurements of the electrical conductivity of the composites based on PP for a range of concentrations of nanosized fillers of various types are presented in Figure 6. Due to the large error of evaluation of electrical resistivity higher than 10^12^ Ohm∙cm with the equipment used we used constant value of 10^12^ Ohm∙cm for every electrical resistivity above this value.

From analysis of the concentration dependences of the electrical conductivity of the composite materials for each type of the filler (Figure 6), the corresponding values of the percolation threshold *c_c_*. were estimated by fitting the curves with Boltzmann sigmoidal function [44]. The results are presented in Table 2.

The obtained from experimental results analysis values of the percolation thresholds for MWCNTs and SWCNTs turned out to be significantly higher than those predicted by theory [45,46] values (Table 2). Such differences are related to the quality of dispersion of filler particles in the polymer matrix for the selected method of composite processing. This conclusion is supported by the electron microscopy data (Figure 3c,d). Particularly noticeable difference for SWCNTs can be explained by the fact that the structure of the SWCNTs powder is complex, since the investigated SWCNTs usually come in form of yarns. At the same time, the obtained value of the percolation threshold for CB was significantly lower than the value theoretically predicted for spherical inclusions [45] (Table 2). This result can be explained by the formation of agglomerates from CB particles with aspect ratio >1 (Figure 1a).

**Figure 7:**
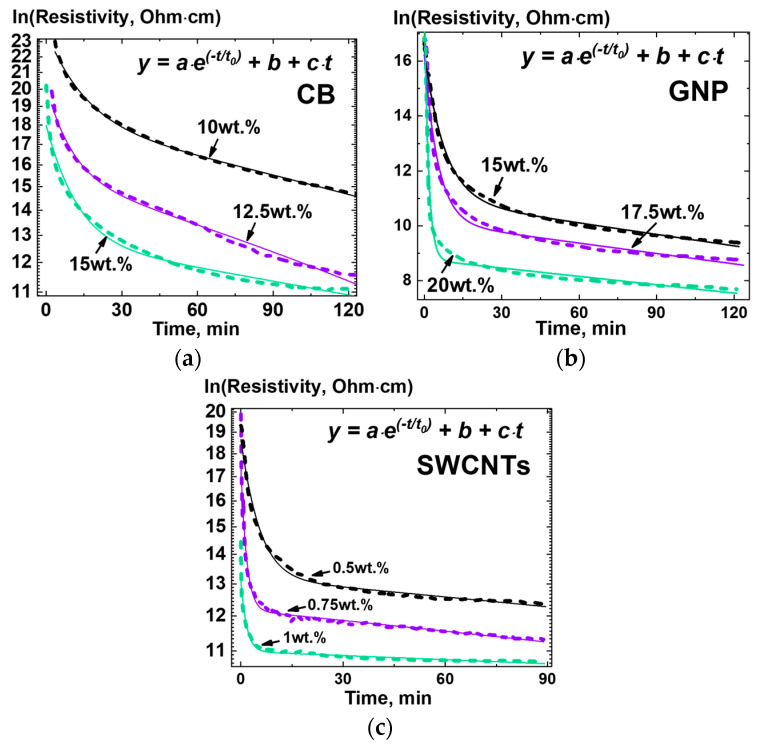
Experimentally obtained dependences of resistivity on time for melts of the composites PP/CB (**a**), PP/GnPs (**b**), and PP/SWCNTs (**c**) with approximation by the relation (4).

At the next stage of the study, the time dependences of the electrical resistivity of composite materials in a melt state were measured for each filler type and for a wide range of the filler content (Figure 7). The measurements were carried out according to the scheme shown in Figure 4, while the geometry of the mold was used to calculate the resistivity. The values of the content of the fillers for each filler type were chosen taking into account the corresponding percolation threshold values.

For all compositions studied, it was found that the resistivity of the composite melts with filler content below the percolation threshold at the start of the thermal treatment was expectedly high. However, at long exposure times a noticeable (>4 decimal orders of magnitude) decrease in the value of the resistivity of the composites was observed for each type of filler studied (Figure 7).

Possibility of thermal destruction of polymer during the heat treatment was investigated by measuring viscosity of the PP before and after holding it in a melt state at 200 °C for 2 h. The results demonstrated that viscosity did not change significantly in the course of the heat treatment (it was in the range of error, after the treatment the viscosity was insignificantly higher—1.5 and 1.6 dl/g before and after heat treatment, respectively).

For all types of polymers and fillers studied in this work, as well as the filler content in composites, it was found that the kinetic curves of resistivity demonstrated the presence of at least two regions—a fast (exponent-like) and a slow. To analyze the obtained dependences, it was decided to use a simple analytical relation containing an exponential part with a certain characteristic decay time *t_0_*. According to this, the following function was chosen:

(4) $$ln(\rho )=a\cdot e^{\frac{-t}{t_{0}}}+b-c\cdot t$$

where ρ is resistivity, *t* is time, *t*_0_ is characteristic time, (*a*, *b*, *c*) are supplementary parameters of the model.

**Figure 8:**
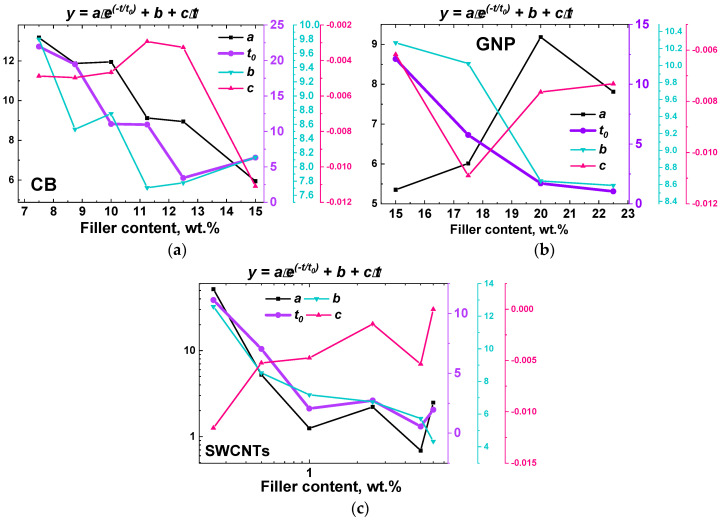
Dependences of the parameters of the equation (4) for the conductivity-time curves (Figure 7) on the filler content for the PP/CB (**a**), PP/GnPs (**b**) and PP/SWCNTs (**c**) composites.

The function (4) was proposed as an alternative to Equation (2), allowing to parametrizing kinetic curves for electrical conductance, obtained experimentally (Figure 7), and find possible correlations of the equation parameters with the parameters of the composite material. It demonstrated the best results in approximating the obtained experimental curves with the minimum number of mutually independent optimized parameters (average adjusted R^2^ ~ 0.98, with the lowest (the worst fitting quality) value ~0.95, and the highest > 0.999). The linear term (*b—c∙t)* can be considered as the first components of the expansion of the exponent with a characteristic decay time much longer than the experiment times. The dependences of the values of the parameters of the relation (4) on the content of the filler in the composites are presented in Figure 8. Standard deviation values are also indicated in Figure 8 for each parameters value, being relatively small in absolute value to distinguish them behind the curves and symbols.

**Figure 9:**
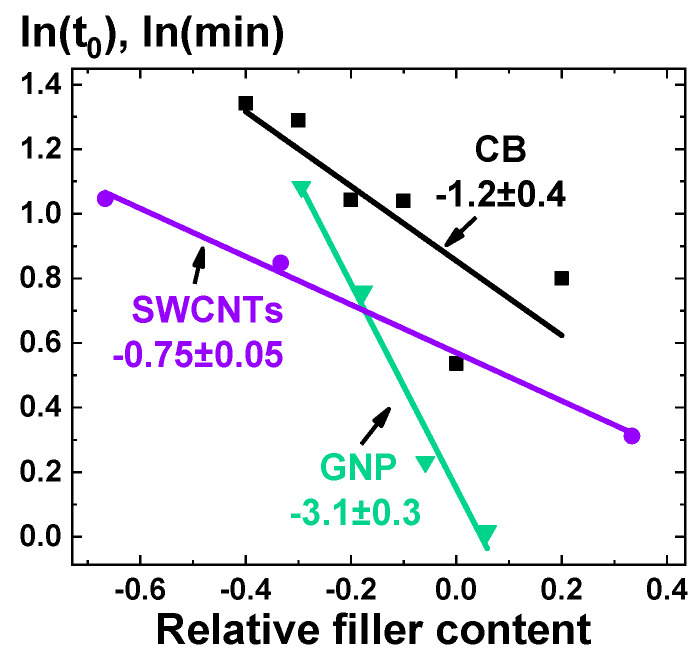
Dependences of the logarithm of the characteristic time *t*_0_ of the fast (exponential) component (4) of the filler segregation kinetics on the relative filler content (5) for PP/CB, PP/GnPs, and PP/SWCNTs composites.

As it can be seen in Figure 8, the only parameter that does not demonstrate any distinctive trends is the parameter *c*, which is relatively small in the absolute value. Analysis of all the parameters can be performed, but to demonstrate a possibility of correlation of the Equation (4) parameters and parameters of the composites, we decided to investigate the parameter *t_0_* only. Thus, the analysis of the model parameter *t_0_* obtained from the approximation of the kinetic curves for composite melts based on PP and containing CB, GnPs, and SWCNTs was performed (Figure 9). To be able to compare the *t_0_* values for composites containing fillers of different types, it was decided to use the relative filler content *c_n_*:

(5) $$c_{n}=\frac{\left(c-c_{с}\right)}{c_{с}},$$

where $c_{с}$—percolation threshold previously determined experimentally (Figure 6, Table 2).

**Figure 10:**
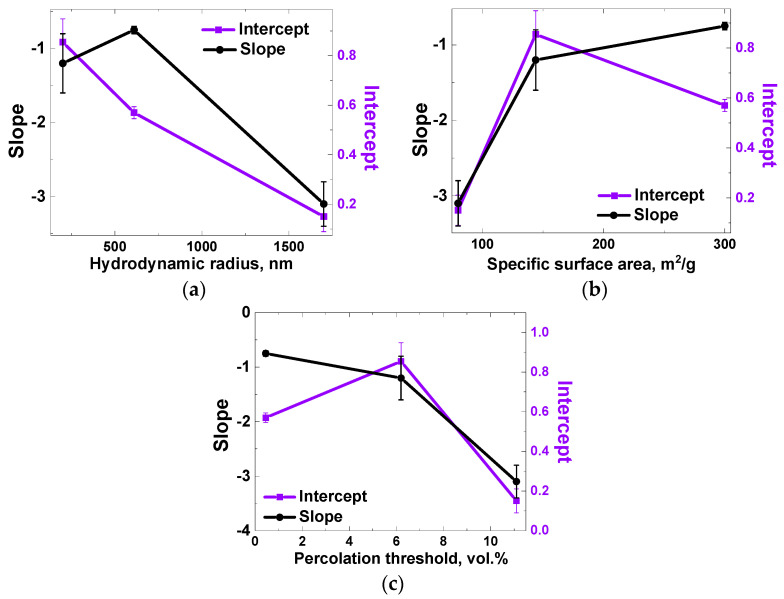
Slopes and intercepts of the quasilinear dependences of the logarithm of the characteristic time *t*_0_ of the fast (exponential) component (4) of the filler segregation kinetics on the relative filler content for PP/CB, PP/GnPs, and PP/SWCNTs composites (Figure 9) versus the filler particles hydrodynamic radius (**a**) and specific surface area (**b**), and the percolation threshold (**c**).

Analysis of the dependences of *ln(t*_0_*)* on *c_n_* made it possible to establish their close to a linear character for concentration values up to values slightly exceeding the percolation thresholds (Table 2). The slopes and intercepts of these linear curves were used as characteristic values for the further analysis of segregation process of the filler particles of a certain geometry (shape). It was possible to obtain a close to linear dependence of the intercept on the calculated hydrodynamic radius for experimentally observed agglomerates of nanoparticles of each type (Figure 10а). The hydrodynamic radius for CB was estimated as an average radius of CB particles agglomerates (~200 nm), which was determined from electron microscopy data (e.g., Figure 3а) according to the method described in ASTM D3849-14a. The hydrodynamic radius of the GnPs was estimated through the radius of gyration $R_{g}$ [47], which for disks equals $R_{g}\;=\;R/\sqrt{2}$, where *R* is the radius of the disk. If the value of 2500 nm is taken as the radius of the GnP particle, then the radius of gyration for it will be ~1700 nm. For SWCNTs, the hydrodynamic radius was estimated using an analytical relation [48], reliability of which was confirmed by the dynamic light scattering method [49]. The ratio used was:

(6) $$R_{h}=\;\frac{l}{ln\left(\frac{l}{d}\right)+0.32},$$

where *l* and *d* are the length and diameter of SWCNTs, respectively.

This result suggests that for large particles the segregation process can be noticeable at short observation times only at relatively high filler concentrations, sufficiently close to the percolation threshold for a given type of composite. Additionally, the dependence of the characteristic time of the segregation process on such parameter as a hydrodynamic radius of a particle may be an evidence of the diffusive nature of the process. Thus, using the estimated value of the hydrodynamic radius of agglomerates of carbon nanoparticles and the measured values of the percolation threshold for the combination polymer/filler/(mixing method), it might possible to predict the characteristic time of evolution of the electrically conductive properties of a composite melt for a given degree of the composite filling.

The use of the correlation dependences of the hydrodynamic radius of nanoparticle agglomerates and the parameters of the relative filler content versus *ln(t*_0_*)* curves is not the only possible way to analyze the filler segregation process. When comparing the slopes of the relative filler content versus *ln(t*_0_*)* curves (Figure 9) with the values of the percolation thresholds and specific surface areas for the corresponding fillers, one can see a monotonicity of the obtained dependences (Figure 10b,c).

**Figure 11:**
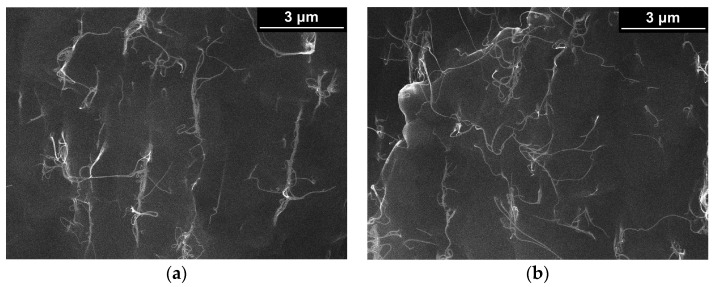

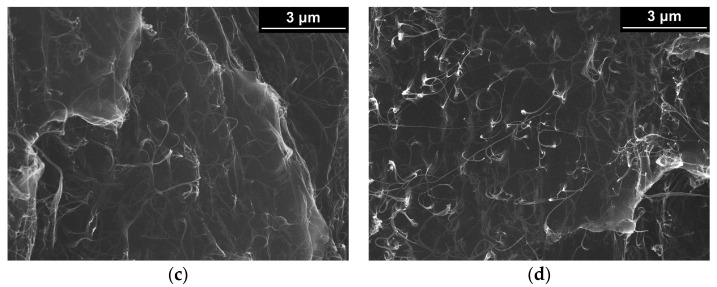
Results of scanning electron microscopy of low-temperature cleavages of the composites (**a**,**b**) PP + 0.5 wt.% SWCNTs and (**c**,**d**) PP + 7.5 wt.% SWCNTs: (**a**,**c**)—the distribution of the filler in the center of the cleavage, (**b**,**d**)—at the edge of the cleavage (surface layer of the initial sample) in the zone up to 30 microns from the edge.

To understand what kind of filler segregation occurs in the bulk of the investigated composites, it was decided to conduct electron microscopy studies of low-temperature cleavages of the PP/SWCNT composites with the filler content lower and higher than the percolation threshold (Table 2). Thorough microscopy studies were performed for composites containing different amounts of the SWCNTs and held in a melt state at 205 °C for 2 h. Detailed investigation of the distribution of the filler showed that it was close to a uniform in the bulk of the composites. At the same time, there was some difference between the patterns obtained near the edge (initially the surface layer of the samples) and in the center of the cleavages. Examples of the microscopy images, obtained for low-temperature cleavages of the composites PP + 0.5 wt.% SWCNTs and PP + 7.5 wt.% SWCNTs, are presented in Figure 11. The images obtained for the profile of the near-surface layer of the composite sample (up to 30 μm from the surface, Figure 11a,c) demonstrate something more similar to percolation, in comparison with the distribution of SWCNTs in the center of the cleavage (Figure 11b,d).

Since the data obtained by electron microscopy does not allow to obtain a complete understanding of the filler distribution in the composites, it was proposed to use a more direct method for determining the contribution of the near-surface layer to the electrical conductivity of the material as a whole. Thus, additional experiments were carried out, consisted of removal of a controlled amount of material from the surface of the PP + 12.5 wt.% CB composite sample cooled to room temperature after being held in a melt state for 2 h. The melt of the composite was cooled in the ice water, after which several layers of material were removed from the flat surface of the sample with a cutter, sequentially increasing the depth of the groove by 10 μm for each pass. After each layer removal, the surface resistance of the composite was measured using a multimeter by a two-probe method with a distance between the electrodes of ~10 mm.

**Figure 12:**
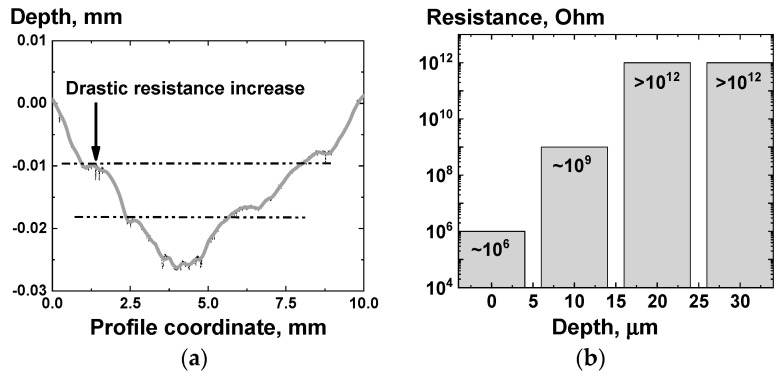
The results of measuring the surface resistance of the composite PP + 12.5 wt.% CB by the two-point method (**b**) before and after removal of the near-surface layer of various thicknesses (**a**).

An example of the results, obtained for one of the investigated composite samples PP + 12.5 wt.% CB, is presented in Figure 12. A profilometer was used to control the depth of the groove on the surface of the composite (Figure 12a). It was found that after removing a layer ~10 μm thick from the surface of the composite, the value of the measured surface resistance of the composite increased by several decimal orders (Figure 12b).

Taking into account the results of the electron microscopy and measurements of surface resistance before and after the removal of thin surface layers, it is possible to formulate a hypothesis about the presence of a near-surface layer enriched with filler particles. The effective thickness of the supposedly formed highly conductive near-surface layer for PP/CB composites can be given an upper estimate of ~10 μm, taking into account that the estimation in this way is still rather rough. Since thicknesses of the samples studied (Figure 4) was high compared to the expected thickness of the enriched surface layer, the usage of the linear component in the Equation (4) is acceptable, considering the times required to reach equilibrium across the whole composite volume.

The analysis of the time dependences of electrical conductivity was also carried out for the composites based on polyethylene and polystyrene (PS) and filled with SWCNTs. The use of PS made it possible to additionally give a qualitative assessment of the possible influence of the existence of the crystalline phase in the polymer at any stage of its processing on the presence of the studied effects of the composite conductance change. For both types of composites, time dependences of electrical conductance were also observed during their stay in a melt state. These dependences were similar to those previously investigated for composites based on PP and were also well approximated using function (4).

**Figure 13:**
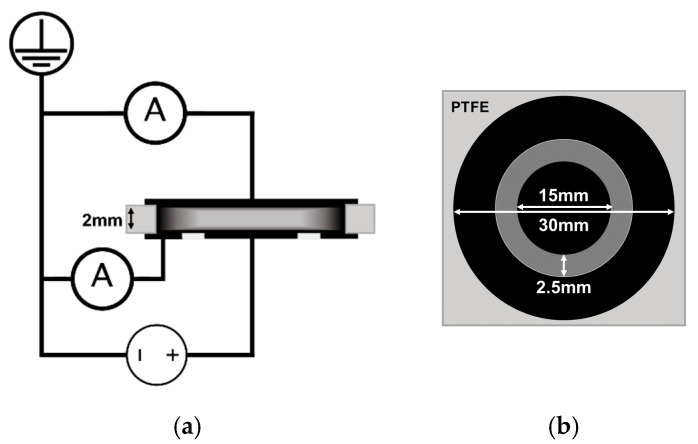
Experimental three-electrode scheme for measurement of the electrical resistance of a composite melt with separation of the surface and bulk components: (**a**)—side view; (**b**)—top view.

The two-electrode scheme for measuring the electrical conductivity of a composite material (Figure 4) does not give a definitive answer on what is the possible impact of the assumed process of migration of nanoparticles to the composite surface on the observed phenomenon of an increase in the electrical conductivity of composites during their stay in a melt state. It was decided to modify the experimental approach of obtaining electrophysical properties of the composite melts. For this purpose, an alternative experimental scheme was developed, based on the ASTM D257-14 standard, which describes methods for measuring simultaneously the surface and volume DC resistance of dielectrics. The developed scheme makes it possible to relatively independently investigate the electroconductive properties of the bulk and near-surface layer of composite material melt samples using two picoammeters simultaneously recording the values of the both flowing currents (Figure 13). The resistivity of the near-surface layer was estimated as the resistivity of the material located between two “effective” concentric cylinders playing the role of electrodes (upper estimate). In the calculations, the thickness of the composite sample was used as the value of the cylinder height. The diameters and the distance between the cylinders were assumed to be equal to the diameters of the annular electrodes pressed against the melt surface and the distance between them, respectively. Foil-clad fiberglass plates with rings etched on the copper foil were used as electrodes.

**Figure 14:**
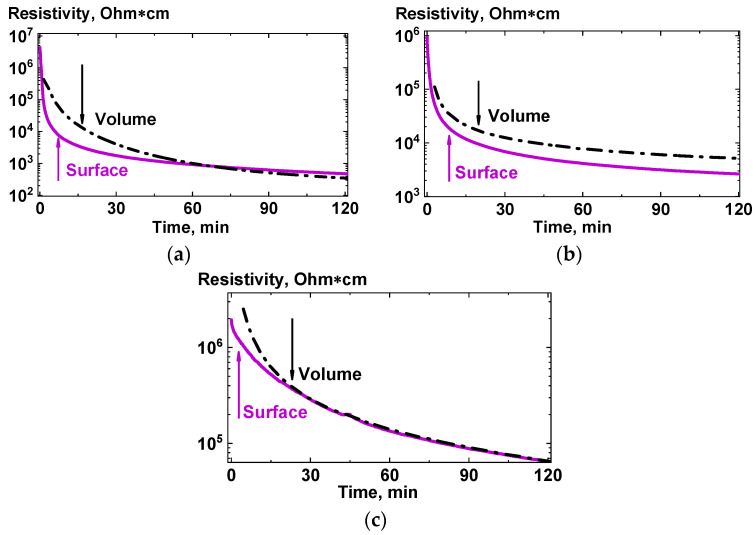
Time dependences of the volume and surface components of the resistivity of PP + 12.5 wt.% CB (**a**), PP + 15 wt.% GnPs (**b**), and PP + 0.5 wt.% SWCNTs (**c**) composites melts. The arrows show the characteristic times for the “fast” regions of the curves.

The obtained electrical resistivity dependences on time, carried out for a wide range of content of each type of filler (CB, GnPs, SWCNTs) in the composites, showed that for any type of filler the kinetic curves for the resistivity of the near-surface layer and the volume of the composite are have noticeable differences, even at relatively high initial filler contents (Figure 14). Considering that the values of the surface component are very rough upper estimates, a high level of surface conductivity can be explained by a high concentration of an electrically conductive filler in a near-surface layer of a certain thickness, which can increase during the process of the composite being in the melt state at a fixed temperature. Unfortunately, due to some artifacts, observed for composites with low content of the fillers, no reliable kinetic curves for the resistivity can be presented at this moment. Although it should be noted that the tendencies, visible for the relatively high contents of the filler, are also present for the composites with a low filler content.

The effect of a rapid increase in electrical conductivity, both of the surface and bulk components, manifested in the initial range of the holding times of the composite in a melt state (Figure 7 and Figure 14), can be explained by the process of formation of contacts between the filler particles located in the near-surface layer and the electrode. Due to the fact that, after mixing in the extruder, the nanoparticles are mechanically enveloped with a polymer layer of a certain thickness, the formation of a contact during pressing may take some time, even if the particle is initially quite close to the surface. At the same time, for high filler concentrations, the observed significant increase in the electrical conductivity of the volume can be associated with the agglomeration of filler particles around the already formed percolating structures. Additionally, particles that come from the bulk can increase concentration of the next particles monolayer, thus increasing effective thickness of the highly conductive surface layer. Due to the diffusion nature of this process, its characteristic times can be very high.

**Figure 15:**
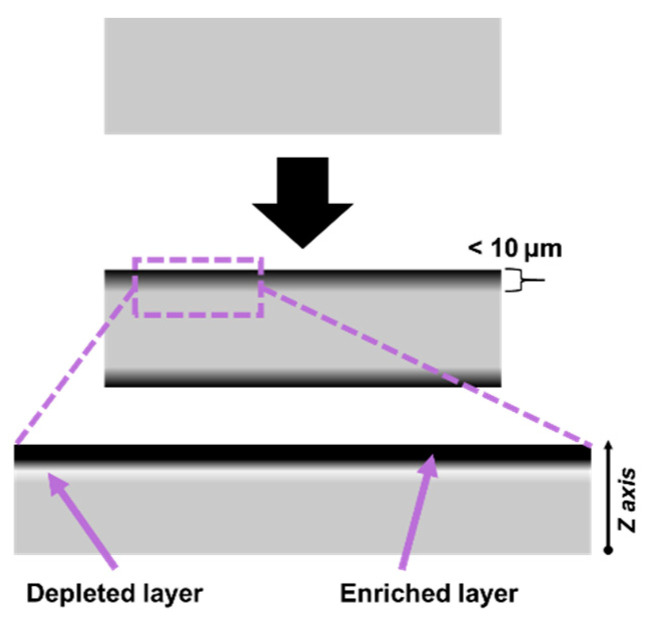
Schematic illustration of the assumed distribution of the electrically conductive filler before (**above**) and after (**below**) holding the composite material in a melt state.

It was noted earlier that for all the measured kinetic curves of electrical conductivity, at least two regions can be distinguished—fast and slow. In addition, one can note a certain systematic delay for the characteristic time of the fast region for bulk component of the conductivity compared with that for the surface component (characteristic times are shown with arrows in Figure 14). This effect can be explained by the presence for certain period of time of a particle-depleted layer located under the enriched layer. A proposed schematic representation of the formation of a near-surface layer enriched with nanoparticles in a composite during its stay in a melt state is shown in Figure 15.

For initial filler (of any type) contents that are noticeably below the percolation threshold, a drop in the measured resistivity of the bulk component with time was observed. It is assumed that this can be caused by agglomeration of nanoparticles in the bulk, accompanied by a decrease in the effective ratio of the geometric sizes of particles, their mobility, and the probability of the formation of an electrically conductive cluster passing through the entire volume of the composite.

The control experiments showed that the effects described above does not depend on the type of material of the contact electrodes (copper, aluminum) and spacers (PTFE, fiberglass), as well as on their presence or absence during the stay the composites in a melt state at a fixed temperature for long periods of time.

### 3.2. Numerical Studies

To obtain a detailed understanding of what is happening in the bulk and at the surface of the composite material during its stay in a melt state depending on various parameters, such as geometry of the nanoparticles, their content, as well as the temperature of the composite and amplitude of the forces of nanoparticle/wall and nanoparticle/nanoparticle interactions, a numerical model of a composite material has been developed that models evolution of the distribution of nanoparticles under conditions similar to the experimental ones. Numerical modeling was carried out using MD methods for composites filled with CNTs (see Section 2.2.3).

The analysis of the results of modeling by MD methods was carried out using histograms of the distribution of CNTs in the simulated cell along the *Z* axis (Figure 5). The initial content of the uniformly distributed filler in the volume, temperature, and the amplitude of the forces of interaction between CNTs and between CNT and the walls were chosen as the main parameters.

**Figure 16:**
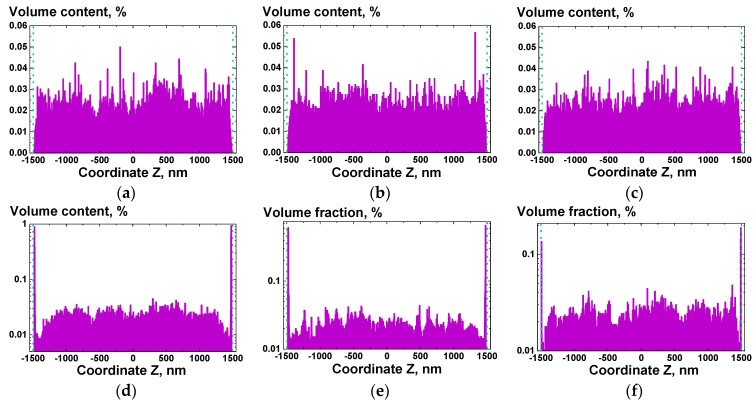
Results of numerical simulation using MD methods for composites containing CNTs with a total volume fraction of 0.5 vol%, for the distribution of the filler along the Z axis at a time step 0 (**a**–**c**) and at a time step 1000 (**d**–**f**) for different amplitudes of interaction forces: (**a**,**d**)—weak CNT/CNT, strong CNT/wall; (**b**,**e**)—strong CNT/CNT, strong CNT/wall; (**c**,**f**)—weak CNT/CNT, weak CNT/wall interactions.

Analysis of the histograms of the filler distribution along the *Z* axis (Figure 16) revealed the effect of the formation of a depleted for a certain period of time layer in the bulk of the composite at a distance of several tens of nanometers from the walls. Depletion was caused by the rapid migration of CNT segments localized near the attractive wall to their surface, causing a sharp increase in the concentration of CNTs in the near-surface layer. This effect is especially noticeable when the CNT/wall interaction significantly exceeds the CNT/CNT interaction (Figure 16a,d). With time, the depleted layer disappeared, accompanied by an increase in the near-surface concentration of CNTs. Depending on the content of the filler in the volume element, the degree of depletion varied, as well as the time of its disappearance. For high content values, the depletion became noticeable for a short period of time, with a small amplitude of the change in the local volume fraction of CNTs. At the same time, at low CNT contents, it took a rather long amount of time for local volume fraction to equalize.

It was also possible to see that in the presence of even a rather weak attractive potential between CNTs at high concentrations of CNTs (>1 wt.%), a competition between the near-surface layer enrichments process and the formation of agglomerates from CNTs occurs. If the CNT/CNT interaction becomes comparable in amplitude to the CNT/wall interaction, the resulting CNT agglomerates in the bulk of the composite no longer allow for the depleted layer to be restored.

All described effects are fully reflected in the obtained experimental data (Figure 14). This allows to say that the numerical method proposed in the work is a handy tool for studying the effect of the filler content, its interaction energy with other particles and with the wall, on the formation of an enriched near-surface layer, which results in an increase in the electrical conductance of the material as a whole.

Additionally, the simulations of the kinetics of the nanoparticles segregation illustrated how different parameters of the system affects the formation of the enriched near-surface layer in the polymer composite melts. Specifically, it was demonstrated that at lower contents of the filler (below 1 wt.% of CNTs) the enrichment process is mostly determined by the strength of the nanoparticle/wall interaction, while at higher contents (above 1 wt.% of CNTs) the most critical parameter is nanoparticle/nanoparticle interaction strength. The interaction forces magnitude also determines how fast the depleted layer forms and disappears, which can be used in the future to correlate the kinetics, obtained using simulations, to the experimental results for different types of polymers. Initial distribution also significantly affects the enrichment, since in the case of agglomeration the effective mass and size of the particles can increase significantly. It is planned to investigate the influence of dispersity and the shape of the nanoparticles on the kinetics and formation of the enriched layer in the future.

## 4. Discussion

In view of the fact that the electroconductive properties of the composite material change during its stay in the melt state, it was concluded that the formation of a saturated layer also occurs in the melt. It was demonstrated that the effect of an increase in conductivity after holding the composite in a melt state is observed for any areas of the surface of the samples for which the resistance was measured. In addition, the disappearance of conductivity after removal of a thin surface layer takes place for any location of the milled on the surface of the sample groove. Thus, it is possible to exclude sedimentation as one of the reasons for the formation of an assumed saturated layer. It is obvious that the redistribution of the filler in the composite without an initial concentration gradient and without external temperature and electromagnetic fields (with the expectation of the absence of the sedimentation effect) can occur only due to the migration of nanoparticles due to the Brownian motion. In this case, the saturation of the near-surface layer should be due to the energy gain obtained by the composite system when the filler particles migrate to the surface. This is true under the assumption that the particles of the fillers used in the work do not have a constant electric charge (there is no Coulomb interaction). In general, if we use the terminology applied to chemical reactions, a spontaneous change in the free energy *G* (Gibbs energy) during the migration of filler particles to the melt surface can be described by the following relation [50]:

(7) $$\Delta G=\;\Delta H-T\Delta S=s\cdot \left[\left(H_{np,s}-H_{np,b}\right)+\;\left(H_{s,b}-H_{s,s}\right)\right]-\;T\;\cdot \left[w_{np}\cdot \left(S_{np,s}-S_{np,b}\right)-\;w_{s}\cdot \left(S_{s,b}-S_{s,s}\right)\right]<0,$$

where *H* is the total enthalpy of the system; *T* is the absolute temperature of the medium; *S* is the total entropy of the system; *s* is the surface area of a nanoparticle that changes the contact with the polymer to contact with the components of the mold or air; $H_{np,s}$ and $H_{np,b}$ are the specific enthalpy (specific surface energy) of the nanoparticle at the boundary and in the bulk of the composite, respectively; $H_{s,s}$ and $H_{s,b}$ are the specific enthalpy (specific surface energy) of the polymer at the boundary and in the bulk of the composite melt, respectively; $w_{np}$ is a certain coefficient of change in the entropy of a nanoparticle when it, or part of it, passes from the bulk to the surface; $S_{np,s}$ and $S_{np,b}$ are the specific enthalpy of the polymer at the boundary and in the bulk of the composite, respectively; $w_{s}$ is a certain coefficient of change in the entropy of the polymer when it passes in a certain amount from the boundaries of the composite melt into its volume; $S_{s,b}$ and $S_{s,s}$ are the specific enthalpy of the polymer at the boundary and in the bulk of the composite, respectively.

For a sufficiently detailed understanding of the reasons for the possible migration of a nanosized filler to the composite surface, it is necessary to consider the potential contribution of each of the components of relation (7).

The enthalpy contribution from the side of nanoparticles interacting with the surface can occur due to the Van der Waals interaction between the components of the mold that confine the melt (hereinafter referred to as the “wall”), or between the nanoparticles themselves. The contribution from the dispersion interaction, for example, for nanotubes crossed at an angle of π/2, can lead to potential well depths of ~30 eV and more [51]. For graphene sheets forming graphite, the exfoliation energy is ~40 meV per atom, which for large contact areas between particles makes a significant contribution compared to $k_{B}T$ [52]. In general, the influence of the Van der Waals interaction can manifest itself in two stages: the primary interaction of the particle and the wall, as well as the subsequent attraction of other particles by this fixed particle. The interaction potential of two particles, which takes into account the dispersion interaction, has a minimum at distances of ~3–4 Å, while the range of action of Van der Waals forces can be tens of nm, although at long distances the Brownian motion begins to dominate. On this basis, it can be assumed that the saturation of the near-surface layer due to the contribution of Van der Waals forces occurs according to a scheme similar to multilayer adsorption.

Since the effect of an increase in the conductivity of the composite, which occurs according to the hypothesis of the migration of the filler to the boundaries of the melt of the composite material also manifests itself in the case when the melt of the composite is in an open mold, thereby coming into contact with a part of its surface with a gas medium, we can say that the Van der Waals interaction with the wall is not necessarily the dominant contribution to the observed process of saturation of the surface layer with nanoparticles.

In order to understand what else can cause the accumulation of particles at the surface, we can consider the enthalpy part associated with the polymer, which plays the role of a medium in which the nanoparticles move. Due to the difference in surface energies (specific surface tension) between the carbon nanoparticles and the polymer, it can be energetically advantageous for the polymer to displace the nanoparticles to the surface, and also bring the particles into contact with each other. The total surface energy benefit Δ*E* can be described by the following relationship [50]:

(8) $$\Delta E=s_{1}\cdot E_{np-s}+s_{2}\cdot E_{np-p}-s_{1}\cdot E_{p-\;s}-s\cdot E_{np-p}<0,\;s_{1}+s_{2}=s,$$

where *s* is the total surface area of the particle; $s_{1}$ is the area of the contact surface of the particle with the boundary of the composite after it emerges on the surface of the melt; $s_{2}$ is the contact area of the particle with the polymer after it emerges on the melt surface; $E_{np-s}$ is the specific surface energy of a nanoparticle on the melt surface; $E_{np-p}$—specific surface energy of the nanoparticle-polymer contact zone; $E_{p-\;s}$—specific surface energy of the polymer zone on the melt surface.

**Figure 17:**
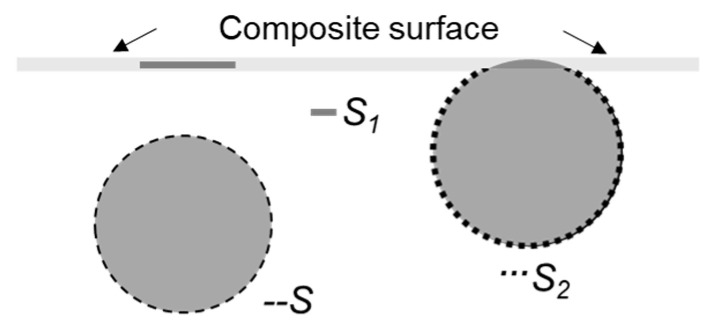
Scheme of changes in the contact surfaces between the polymer, nanoparticle and the melt boundary of the composite when the nanoparticle migrates to the melt boundary.

An illustration of relation (8) is given in Figure 17.

Using the literature data [53,54], one can estimate the value of the energy gain per unit surface. If to take the specific surface area of 20 erg/cm^2^ for the PP at 200 °C, and the value of 5 erg/cm^2^ for a carbon nanoparticle, then when a nanoparticle site with area of 1 nm^2^ comes in contact with the melt boundary, an energy gain of ~0.1 eV >> $k_{B}T$ is obtained. For MWCNTs NC7000, the total surface area of which is ~1.2 × 10^5^ nm^2^, the energy gain can be quite significant even when a small part of MWCNT reaches the melt surface.

Such reasoning about the contribution of enthalpy components makes it possible to qualitatively explain the dependences of the slope and intercept of the quasi-linear concentration dependence of the logarithm of the characteristic time of the fast segregation process of the filler in composites based on PP, depending on the percolation threshold and specific surface area (Figure 10b,c). Taking into account the direct relationship between the specific surface area and surface energy of a particles, one can understand the increase in the sensitivity of the kinetics of the saturation of the near-surface layer to changes in the filler content (Figure 10b). At the same time, the percolation threshold value also plays an important role, since at high filler concentrations, a greater amount of polymer will be in contact with nanoparticles, which may explain a kinetics accelerated response to the addition of the filler.

In the case of the entropy contribution, from the point of view of the entropy of nanoparticles, we can confidently speak of the unfavorable location of the nanoparticle at the melt boundary of the composite, compared to its localization in the bulk due to the loss of one degree of freedom of translational motion. For anisotropic particles, the energy losses can be even greater, especially for particles such as CNTs, which, in addition to the rotational degrees of freedom as a whole, have a certain equilibrium conformation due to close to independent motions of individual parts of the CNT.

The entropy contribution to relation (8) from the side of the polymer can be more significant than the contribution of nanoparticles, due to the greater mobility of polymer molecules, as well as a larger number of polymer particles per unit volume. In addition to the gain in entropy when passing from the melt surface to the bulk of the composite, the polymer also gains in entropy when replacing the polymer/nanoparticle interaction with the polymer/polymer and nanoparticle/wall. Moreover, due to the presence of an equilibrium conformation (statistical coil) in the polymer chain, which the macromolecule tries to maintain (isothermal conditions), it becomes beneficial for the polymer to displace nanoparticles to the surface. This interaction between the polymer, nanoparticles and the wall can be described in terms of the theory of depletion forces [13,14,15].

Depletion forces between filler particles in the polymer melt are quite strong, and, at the same time, it can act at relatively long range. To evaluate the interaction force amplitude between two particles or a particle and a wall in a polymer melt, the radius of gyration of the polymer chain can be used as the characteristic length of the interaction. Estimates show that for a molecular weight of PP ~300,000, corresponding to the melt flow index of the PP used in this work, the characteristic length of the depletion interaction is ~20 nm, which is comparable to the sizes of individual filler nanoparticles. While, according to [13], when considering polymer melts with colloidal particles distributed in the polymer volume, the range of action of the “depletion” interaction is small (~1 nm), its contribution to the effect of nanoparticle adsorption on the surface or nanoparticle agglomeration can be significant.

Taking into account that between two spherical particles depletion force amplitude is ~2 times weaker than the force amplitude between a spherical particle and a flat wall [13,55], one can expect an initial sharp increase in conductivity in the surface layer due to closely spaced filler particles prevailing over their agglomeration. This is also true for Van der Waals interactions. Based on general considerations, agglomeration of the filler due to such interactions can lead to an increase in the electrical conductivity of the composite volume only in the case of initially achieved percolation.

Miura et al. [56] described in detail the analytical form of the adsorption process, in which the limiting stage of diffusion takes place. The following relation was obtained for the time dependence of the degree of filling of the surface monolayer Γ:

(9) Γ(t)=Γm−Γm·exp(−2·c0ΓmDtπ),

where *t* is time; $\Gamma_{m}$—maximum surface coverage; *с*_0_ is the concentration of the adsorptive in the volume; *D* is the diffusion coefficient for the adsorptive.

Taking into account that the electrical conductivity has a nonlinear relationship with the filler concentration [45], the nonlinear dependence of the filler concentration on the surface on time (9), exacerbated by the multilayer nature of the adsorption process, as well as the nontriviality of the particle shape of some fillers, one can explain why the use of characteristic times obtained by multiple logarithms of the kinetic dependences of the electrical conductivity of composite materials makes it possible to obtain correlation dependences of these times with different parameters of the filler and its distribution (Figure 10).

Summing up the results, it can be concluded that the analysis of the time dependence of electrical conductance can be a useful tool for characterization of evolution in time of the spatial distribution of the filler particles at the microscopic level. The polymer composites obtained and studied in this work with assumingly different characteristics of the surface layer and the bulk can be potentially used, e.g., as (semi)conductors with an insulated substrate made of the same material for various areas of applications in industry.

Although the results of the microscopy studies (Figure 11), experiments with edging of the surface layer of the composite samples (Figure 12), or the results obtained using three electrode measurements scheme (Figure 13) do support our assumption of the presence of the effect of the polymer composite melt near-surface layer enrichment with nanoparticles, additional experimental and numerical data is planned to be obtained before the effect can be claimed with absolute certainty.

## Figures and Tables

**Table 1:** The formulation of composites.

Filler Type	Filler Content, wt.%	PP Content, *c_c_*, wt.%	Sample Code
CB	12.5	87.5	PP + 12.5 wt.% CB
	15	85	PP + 15 wt.% CB
GnPs	10	90	PP + 10 wt.% GnPs
MWCNTs	1	99	PP + 1 wt.% MWCNTs
	7.5	92.5	PP + 7.5 wt.% MWCNTs
SWCNTs	0.5	99.5	PP + 0.5 wt.% SWCNTs
	7.5	92.5	PP + 7.5 wt.% SWCNTs

**Table 2:** Percolation thresholds for PP composites filled with various fillers, determined from the experimental data and the theory.

Filler	Experimental, *c_c_*, wt.%	Theoretical, *c_c_*, wt.%
CB	14 ± 1	35
GnPs	22 ± 1	20
MWCNTs	3 ± 0.5	1.1
SWCNTs	1 ± 0.2	0.09

## Data Availability

The datasets used during this study are available from the corresponding author on reasonable request.

## References

[1] Bose S., Özdilek C., Leys J., Seo J.W., Wübbenhorst M., Vermant J., Moldenaers P. (2010). Phase separation as a tool to control dispersion of multiwall carbon nanotubes in polymeric blends. ACS Appl. Mater. Interfaces.

[2] Tang Y., Lewin M., Pearce E.M. (2006). Effects of annealing on the migration behavior of pa6/clay nanocomposites. Macromol. Rapid Commun..

[3] Tao F., Nysten B., Baudouin A.-C., Thomassin J.-M., Vuluga D., Detrembleur C., Bailly C. (2011). Influence of nanoparticle–polymer interactions on the apparent migration behaviour of carbon nanotubes in an immiscible polymer blend. Polymer.

[4] Xiong Z.-Y., Wang L., Sun Y., Guo Z.-X., Yu J. (2013). Migration of MWCNTs during melt preparation of ABS/PC/MWCNT conductive composites via PC/MWCNT masterbatch approach. Polymer.

[5] Alig I., Lellinger D., Dudkin S.M., Pötschke P. (2007). Conductivity spectroscopy on melt processed polypropylene–multiwalled carbon nanotube composites: Recovery after shear and crystallization. Polymer.

[6] Alig I., Skipa T., Engel M., Lellinger D., Pegel S., Pötschke P. (2007). Electrical conductivity recovery in carbon nanotube–polymer composites after transient shear. Phys. Status Solidi.

[7] Deng H., Skipa T., Zhang R., Lellinger D., Bilotti E., Alig I., Peijs T. (2009). Effect of melting and crystallization on the conductive network in conductive polymer composites. Polymer.

[8] Heinrich G., Costa F.R., Abdel-Goad M., Wagenknecht U. (2005). Structural kinetics in filled elastomers and PE/LDH composites. Kautschuk Gummi Kunststoffe.

[9] Fournier J., Boiteux G., Seytre G., Marichy G. (1997). Percolation network of polypyrrole in conducting polymer composites. Synth. Met..

[10] Pegel S., Pötschke P., Petzold G., Alig I., Dudkin S.M., Lellinger D. (2008). Dispersion, agglomeration, and network formation of multiwalled carbon nanotubes in polycarbonate melts. Polymer.

[11] Salehiyan R., Ray S.S. (2019). Tuning the conductivity of nanocomposites through nanoparticle migration and interface crossing in immiscible polymer blends: A review on fundamental understanding. Macromol. Mater. Eng..

[12] Teng C.-Y., Sheng Y.-J., Tsao H.-K. (2017). Surface segregation and bulk aggregation in an athermal thin film of polymer–nanoparticle blends: Strategies of controlling phase behavior. Langmuir.

[13] Lekkerkerker H.N., Tuinier R., Metzler J.B. (2011). Colloids and the Depletion Interaction.

[14] Sukenik S., Sapir L., Harries D. (2013). Balance of enthalpy and entropy in depletion forces. Curr. Opin. Colloid Interface Sci..

[15] Edwards T.D., Bevan M.A. (2012). Depletion-mediated potentials and phase behavior for micelles, macromolecules, nanoparticles, and hydrogel particles. Langmuir.

[16] Grekhov A.M., Eremin Y.S. (2015). Influence of carbon-nanotube concentration in chloroform on the kinetics of agglomeration and sedimentation. Nanotechnol. Russia.

[17] Blythe T., Bloor D. (2008). Electrical Properties of Polymers.

[18] Sahimi M. (1994). Applications of Percolation Theory.

[19] Stauffer D., Aharony A., Redner S. (1993). Introduction to percolation theory. Phys. Today.

[20] Kirkpatrick S. (1973). Percolation and conduction. Rev. Mod. Phys..

[21] Zhang Q., Zhang B.-Y., Wang W.-J., Guo Z.-X., Yu J. (2018). Highly efficient electrically conductive networks in carbon-black-filled ternary blends through the formation of thermodynamically induced self-assembled hierarchical structures. J. Appl. Polym. Sci..

[22] Katada A., Konishi Y., Isogai T., Tominaga Y., Asai S., Sumita M. (2003). Dynamic percolation phenomenon of poly (methyl methacrylate)/surface fluorinated carbon black composite. J. Appl. Polym. Sci..

[23] Wu G., Asai S., Sumita M. (2002). Carbon black as a self-diagnosing probe to trace polymer dynamics in highly filled compositions. Macromolecules.

[24] Wu G., Asai S., Zhang C., Miura T., Sumita M. (2000). A delay of percolation time in carbon-black-filled conductive polymer composites. J. Appl. Phys..

[25] Zhao Y., Byshkin M., Cong Y., Kawakatsu T., Guadagno L., De Nicola A., Yu N., Milano G., Dong B. (2016). Self-assembly of carbon nanotubes in polymer melts: Simulation of structural and electrical behaviour by hybrid particle-field molecular dynamics. Nanoscale.

[26] Gumerov R.A., Rudov A.A., Richtering W., Möller M., Potemkin I.I. (2017). Amphiphilic arborescent copolymers and microgels: From unimolecular micelles in a selective solvent to the stable monolayers of variable density and nanostructure at a liquid interface. ACS Appl. Mater. Interfaces.

[27] Naebe M., Shirvanimoghaddam K. (2016). Functionally graded materials: A review of fabrication and properties. Appl. Mater. Today.

[28] Lebedev O.V., Bogdanova O.I., Goncharuk G.P., Ozerin A.N. (2019). Tribological and percolation properties of polypropylene/nanodiamond soot composites. Polym. Polym. Compos..

[29] Abbasi H., Antunes M., Velasco J.I. (2019). Recent advances in carbon-based polymer nanocomposites for electromagnetic interference shielding. Prog. Mater. Sci..

[30] PP H030 GP/3. http://www.sibur-int.com/upload/documents/TDSH030GP3v14.pdf.

[31] P267-E. https://studylib.ru/doc/2305578/e-lektroprovodnyj-tehnicheskij-uglerod-p-267-e---p-268.

[32] Graphene Supermarket. https://graphene_supermarket.com.

[33] Nanocyl. https://www.nanocyl.com/product/nc7000/.

[34] Tuball. https://tuball.com/en/about-tuball.

[35] Krestinin A.V., Dremova N.N., Knerel’Man E.I., Blinova L.N., Zhigalina V.G., Kiselev N.A. (2015). Characterization of SWCNT products manufactured in Russia and the prospects for their industrial application. Nanotechnol. Russ..

[36] LAMMPS. http://lammps.sandia.gov.

[37] Plimpton S. (1995). Fast parallel algorithms for short-range molecular dynamics. J. Comput. Phys..

[38] Frenkel D., Smit B., Ratner M.A. (1997). Understanding molecular simulation: From algorithms to applications. Phys. Today.

[39] Schneider T., Stoll E. (1978). Molecular-dynamics study of a three-dimensional one-component model for distortive phase transitions. Phys. Rev. B.

[40] Flory P.J. (1989). Statistical Mechanics of Chain Molecules.

[41] Lee H.S., Yun C.H., Kim H.M., Lee C.J. (2007). Persistence length of multiwalled carbon nanotubes with static bending. J. Phys. Chem. C.

[42] Everaers R., Ejtehadi M.R. (2003). Interaction potentials for soft and hard ellipsoids. Phys. Rev. E.

[43] And K.A.F., Qin Y. (2006). Molecular-dynamics simulation of colloidal nanoparticle forces. Ind. Eng. Chem. Res..

[44] Rahaman M., Aldalbahi A., Govindasami P., Khanam N.P., Bhandari S., Feng P., Altalhi T. (2017). A new insight in determining the percolation threshold of electrical conductivity for extrinsically conducting polymer composites through different sigmoidal models. Polymers.

[45] Lisunova M., Mamunya Y., Lebovka N., Melezhyk A. (2007). Percolation behaviour of ultrahigh molecular weight polyethylene/multi-walled carbon nanotubes composites. Eur. Polym. J..

[46] Powell M.J. (1979). Site percolation in randomly packed spheres. Phys. Rev. B.

[47] Capuani F., Pagonabarraga I., Frenkel D. (2006). Lattice-Boltzmann simulation of the sedimentation of charged disks. J. Chem. Phys..

[48] Nair N., Kim W.-J., Braatz A.R.D., Strano M.S. (2008). Dynamics of surfactant-suspended single-walled carbon nanotubes in a centrifugal field. Langmuir.

[49] Panalytical M. Combining Dynamic Light Scattering and Raman Spectroscopy to Characterize Single Wall Carbon Nanotubes (SWNTs). https://www.azonano.com/article.aspx?ArticleID=4133.

[50] Subramanian M.N. (2017). Polymer Blends and Composites: Chemistry and Technology.

[51] Zhbanov A.I., Pogorelov E.G., Chang Y.-C. (2010). Van der Waals interaction between two crossed carbon nanotubes. ACS Nano.

[52] Chiou Y.-C., Olukan T.A., AlMahri M.A., Apostoleris H., Chiu C.H., Lai C.-Y., Lu J.-Y., Santos S., Almansouri I., Chiesa M. (2018). Direct measurement of the magnitude of the van der waals interaction of single and multilayer graphene. Langmuir.

[53] Lewin M., Frank R., Mey-Marom A. (2005). Surface free energies of polymeric materials, additives and minerals. Polym. Adv. Technol..

[54] Asai S., Sakata K., Sumita M., Miyasaka K. (1992). Effect of interfacial free energy on the heterogeneous distribution of oxidized carbon black in polymer blends. Polym. J..

[55] Chervanyov A.I. (2011). Depletion forces acting on nanoparticles in confined polymer systems: Potential theory. Phys. Rev. E.

[56] Miura T., Seki K. (2015). Diffusion influenced adsorption kinetics. J. Phys. Chem. B.

